# MLGL-MP: a Multi-Label Graph Learning framework enhanced by pathway interdependence for Metabolic Pathway prediction

**DOI:** 10.1093/bioinformatics/btac222

**Published:** 2022-06-27

**Authors:** Bing-Xue Du, Peng-Cheng Zhao, Bei Zhu, Siu-Ming Yiu, Arnold K Nyamabo, Hui Yu, Jian-Yu Shi

**Affiliations:** School of Life Sciences, Northwestern Polytechnical University, Xi’an 710072, China; School of Life Sciences, Northwestern Polytechnical University, Xi’an 710072, China; School of Life Sciences, Northwestern Polytechnical University, Xi’an 710072, China; Department of Computer Science, The University of Hong Kong, Hong Kong 999077, China; School of Computer Science, Northwestern Polytechnical University, Xi’an 710072, China; School of Computer Science, Northwestern Polytechnical University, Xi’an 710072, China; School of Life Sciences, Northwestern Polytechnical University, Xi’an 710072, China

## Abstract

**Motivation:**

During lead compound optimization, it is crucial to identify pathways where a drug-like compound is metabolized. Recently, machine learning-based methods have achieved inspiring progress to predict potential metabolic pathways for drug-like compounds. However, they neglect the knowledge that metabolic pathways are dependent on each other. Moreover, they are inadequate to elucidate why compounds participate in specific pathways.

**Results:**

To address these issues, we propose a novel Multi-Label Graph Learning framework of Metabolic Pathway prediction boosted by pathway interdependence, called **MLGL-MP**, which contains a compound encoder, a pathway encoder and a multi-label predictor. The compound encoder learns compound embedding representations by graph neural networks. After constructing a pathway dependence graph by re-trained word embeddings and pathway co-occurrences, the pathway encoder learns pathway embeddings by graph convolutional networks. Moreover, after adapting the compound embedding space into the pathway embedding space, the multi-label predictor measures the proximity of two spaces to discriminate which pathways a compound participates in. The comparison with state-of-the-art methods on KEGG pathways demonstrates the superiority of our MLGL-MP. Also, the ablation studies reveal how its three components contribute to the model, including the pathway dependence, the adapter between compound embeddings and pathway embeddings, as well as the pre-training strategy. Furthermore, a case study illustrates the interpretability of MLGL-MP by indicating crucial substructures in a compound, which are significantly associated with the attending metabolic pathways. It is anticipated that this work can boost metabolic pathway predictions in drug discovery.

**Availability and implementation:**

The code and data underlying this article are freely available at https://github.com/dubingxue/MLGL-MP.

## 1 Introduction

**Fig. 1.**
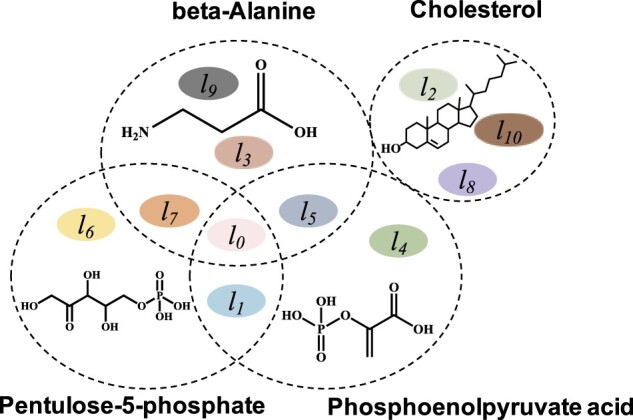
Illustration of compounds and their metabolic pathways. Labels *l_0_*∼*l_10_* represent different types of metabolic pathways. For example, beta-Alanine is metabolized by five pathways, labeled as *l_0_*, *l_3_*, *l_5_*, *l_7_* and *l_9_*. Among them, pathway *l_7_* is shared by Pentulose-5-phosphate and beta-Alanine while pathway *l_5_* is shared by Phosphoenolpyruvate acid and beta-Alanine. Especially, pathway *l_0_* is commonly shared by beta-Alanine, Pentulose-5-phosphate and Phosphoenolpyruvate acid. The list of metabolic pathway names can be found in Table 1

Enzymes catalyze drug or drug-like compounds into their metabolites, which differ significantly from these compounds themselves (Zhang and Tang, 2018). As a complex biotransformation, a compound metabolic pathway contains a set of interlocking enzymatic reactions (Jia *et al.*, 2020a). In drug discovery, it matters to identify what metabolic pathways a compound attends in the stage of lead compound optimization (Baranwal *et al.*, 2020; Cho *et al.*, 2010; Sankar *et al.*, 2017). More importantly, there is a crucial need in drug design to understand why compounds attend specific metabolic pathways. However, since a compound (e.g. beta-Alanine) would attend one or more pathways (Fig. 1), biological assays are always costly and time-consuming to identify pathways among a vast set of pathway combinations. In recent years, computational methods, especially machine learning-based methods, are promising to predict possible metabolic pathways rapidly for given compounds (Baranwal *et al.*, 2020; Zhu *et al.*, 2021).

Former machine learning-based methods can be roughly categorized into network-based and classification-based. Network-based methods generally construct certain interaction networks and leverage network propagation algorithms to infer potential pathways for compounds. For example, Hu *et al.* (2011) constructed a network of chemical–chemical interactions (CCIs) to predict the association of a query compound to 11 kinds of metabolic pathways. As its extension, Gao *et al.* (2012) integrated three networks, involving CCIs, protein–protein interactions (PPIs) and chemical–protein interactions (CPIs) to predict metabolic pathways. Very recently, Zhu *et al.* (2021) proposed a heterogeneous network involving chemicals, enzymes, CCI, CPI and PPI information, where nodes are chemicals or enzymes, and edges are interactions between nodes. However, the major limitation of network-based methods cannot process the compounds which are isolated in the network.

Classification-based methods leverage the technique of multi-label learning to infer potential metabolic pathways. Usually, there are three kinds of multi-label learning strategies. The first technique converts a multi-label classification into one or more binary classifications. For example, taking SVMs the binary classifier, Fang and Chen (2017) set compound-pathway pairs as samples where positive samples are validated pairs and negative samples are unknown pairs. Guo *et al.* (2018) first construct seven compound association networks from KEGG and STITCH. Then, they apply Random Walk with Restart to generate network embeddings, which further were fused as the final compound features. Last, they train a set of pathway-specific binary SVMs. As an extension of Guo *et al.* (2018), Jia *et al.* (2020) recently use random forest (RF) as basic classifiers for accommodating more sub-types of pathways. However, these methods ignore the relationship between labels and aggregate the imbalance between positive and negative samples by generating many negative samples.

The second sort uses a random k-label sets (RAKEL) (Tsoumakas *et al.*, 2007) to treat each kind of label combinations as a new label. Thus, it turns the multi-label classification into a multi-class classification. Compared to the previous binary classification, it provides the co-occurrence of multiple labels. For example, iMPT-FRAKEL (Jia *et al.*, 2020) encodes compounds by fingerprints and leverages a random k-label sets algorithm to tackle the multi-label classification by SVMs and RF. However, these methods also face the imbalance issue where new combined labels usually account for few samples.

The last one directly performs a direct multi-label classification by deep learning. For example, Baranwal *et al.* (2020) and Yang *et al.* (2020) utilized graph neural networks (GNNs) [i.e. graph convolutional network (GCN) and GATs, respectively] to extract compound features based on 2D compound graphs and concatenated them with additional features derived from fingerprints (e.g. MACCS) and molecular properties (e.g. the number of aromatic rings, molecular weight and log *P*) as the final features. However, these methods ignore the dependence between pathways.

In summary, although existing methods have achieved inspiring performance in metabolic pathway prediction, they neglect the dependence between pathways (i.e. pathway crosstalk in terms of biology). For example, both Lipid Metabolism (LM) and Carbohydrate Metabolism (CM) are affected by Metabolism of Cofactors and Vitamins (CVM). Deficiencies of vitamin B1, folic acid and vitamins B6 and B12 (in CVM) lead to a significant increment of lipid deposits in the aorta (in LM) (Kalyesubula *et al.*, 2021; McNeil *et al.*, 2012). In addition, The B group vitamins help convert carbohydrates into energy in CM (Calderón-Ospina and Nava-Mesa, 2020). Moreover, such a pathway interdependence is asymmetric due to upstream/downstream relationships between pathways (Yan *et al.*, 2020). Therefore, the characterization of asymmetric interdependences among pathways would enhance the prediction task in the context that a compound participates in uncertain numbers of metabolic pathways.

Furthermore, existing computational methods are inadequate to interpret why a compound attends a specific metabolic pathway (Baranwal *et al.*, 2020; Yang *et al.*, 2020). In fact, metabolic pathways are usually related to the presence of certain chemical substructures. For example, amino and carboxylic substructures play an important role in binding to enzymes in Amino Acid Metabolism (Lopez and Mohiuddin, 2021). Therefore, the capture of crucial substructures (possibly revealing functional groups), will help reveal the mechanism of a compound metabolized by enzymes.

To address the above issues (pathway interdependence and interpretability), we develop a Multi-Label Graph Learning framework enhanced by pathway interdependence for Metabolic Pathway prediction (MLGL-MP). This end-to-end framework contains a compound encoder, a pathway encoder and a multi-label predictor. The compound encoder learns compound embedding representations based on molecular graphs, while the pathway encoder learns pathway interdependence embeddings. The multi-label prediction discriminates which pathways a compound attends based on two kinds of embeddings. Overall, the main contributions of our MLGL-MP are as follows.

1. It provides an interpretable manner to indicate crucial compound substructures which are significantly associated with metabolic pathways.
2. By capturing the pathway interdependence, it significantly improves the characterization of the relevance between compounds and their metabolic pathways.
3. It proposes a direct multi-pathway prediction approach by measuring the proximity between compounds and metabolic pathways in a common embedding space.

## 2 Materials and methods

### 2.1 Problem formulation and model construction

**Fig. 2.**
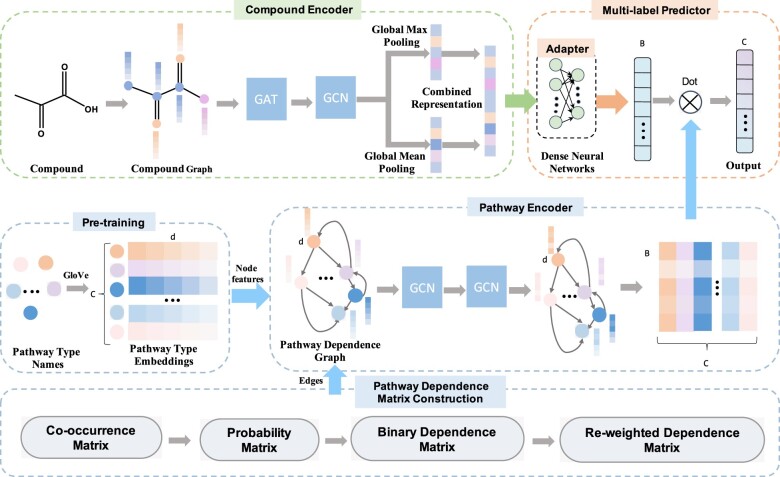
The overall framework of MLGL-MP for multi-label metabolic pathway prediction. It is an end-to-end learning model, which contains a compound encoder, a pathway encoder and a multi-label predictor. The compound encoder generates compound embeddings based on molecular graphs by the composite of a GAT and a GCN. The pathway encoder generates pathway embeddings by two-layer GCNs on a pathway dependence graph, where nodes are pathways, node features are obtained by a pre-training strategy and edges are asymmetric pathway dependences. The multi-label predictor directly discriminates the metabolic pathways of given compounds by the proximity of pathway embeddings and compound embeddings

Given *m* compounds $M=\left\{c_{i},i=1,\ldots ,m\right\}$ and a list of metabolic pathways $T=\left\{t_{c},c=1,\ldots ,C\right\}$. Suppose that a compound $c_{i}$ is assigned with a set of attending pathways $T_{i}\subseteq T$. The task is to predict the pathway set $T_{n}$ of a newly coming compound $c_{n}$, where $T_{n}\subseteq T$. The prediction can be modeled as a problem of multi-label learning, which learns a function mapping $F:M\to 2^{P}$. For this task, we design a multi-label graph learning framework, which contains a compound encoder, a pathway encoder and a multi-label predictor (shown in Fig. 2).

### 2.2 Compound encoder

The compound encoder, adopting a two-layer GNN architecture, learns compound embedding representations by utilizing molecule graphs. Its first layer is a GAT (Veličković *et al.*, 2017), which capture the importance of chemical bonds to pathways. The second layer is a GCN (Kipf and Welling, 2017), which further extracts atom features by aggregating neighboring atom features and is followed by a global pooling layer (Nguyen *et al.*, 2021) to generate compound embeddings.

According to chemical structure, each compound c is represented as a molecule graph G=(V,E), where *V* is the set of *N* atoms and *E* is a set of chemical bonds. Let $A\in R^{N\times N}$ (*N=|V|*) be its adjacency matrix, in which *a_ij_ *= 1 indicates the occurring bond between atom *i* and atom *j*, and *a_ij_* = 0 indicates no bond. Here, each node *v_i_* (atom) is initially represented by a *q*-dimensional binary feature vector $h_{i}\in R^{q}$. As suggested in Nguyen *et al.* (2021), the initial node features typically include the atom symbol, the number of adjacent atoms, the number of adjacent hydrogens, the implicit value of the atom and the atom occurrence in an aromatic structure.

First of all, for each atom $v_{i}$ in the molecular graph G of compound *c*, the GAT layer updates its features by aggregating the features of its neighboring atoms. The aggregation is implemented by a shared self-attention operation *a*: $R^{s}\times R^{s}\to R$, which defines the importance of its neighboring atom $v_{j}$ to atom $v_{i}$ as follows:

(1) $$e_{ij}=a\left(Wh_{i},Wh_{j}\right).$$

Here, the learnable weight matrix $W\in R^{s\times q}$ accounts for a linear transformation from input features into higher-level features, where *s* is the dimension of updated atom features. Then, the updated features $h_{i}^{'}$ of atom *v_i_* can be defined by

(2) $$h_{i}^{'}=\sigma \left(\sum_{j\in N_{i}}\;\alpha_{ij}Wh_{j}\right),$$

where $\alpha_{ij}\;$= softmax_*j*_ ($e_{ij})\;$is the normalized attention coefficient and σ(·) is a non-linear activation function (i.e. ReLU). The normalization of attention coefficients makes themselves comparable among different nodes.

Furthermore, a multihead attention is adopted to enhance the expression of the attention layer. Specifically, *K* independent attention mechanisms are performed in parallel and their features are concatenated as the updated features $h_{i}^{A}$ of atom $v_{i}$ as follows:

(3) hiA=K∥k=1σ∑j∈Ni αijkWkhj,

where $h_{i}^{A}\in R^{Ks},\;Ks$ is the dimension of updated atom features, *K* is the number of attention heads, and ∥ is the concatenation operation of vectors. It is remarkable that the average of ${\{\alpha }_{ij}^{k}\}$ accounts for the importance of the chemical bond between atom *v_i_* and atom *v_j_*.

After that, the GCN layer following the GAT layer further updates atom features by emphasizing the topology of molecule graph. According to the propagation rules of GCN, the updated features $\left\{h_{i}^{C}\in R^{G},i=1,\ldots ,N\right\}$ of atoms of compound *c* is determined by the following matrix form:

(4) $$H^{c}=\sigma \left({\tilde{D}}^{-\frac{1}{2}}\tilde{A}{\tilde{D}}^{-\frac{1}{2}}H^{A}W^{A}\right),$$

where $H^{A}\in R^{N\times Ks}$ is the GAT-based feature matrix stacked by $\left\{h_{i}^{A}\in R^{Ks},i=1,\ldots ,N\right\}$, $H^{c}\in R^{t}$ is the GCN-updating feature matrix stacked by the updated atom feature vector $h_{i}^{c}\in R^{t}$, $W^{A}{\in R}^{Ks\times G}$ is the weight matrix in the GCN layer, $\tilde{A}=A+I_{N}$, $I_{N}$ is the identity matrix, **D**  $\in R^{N\times N}$ is the degree matrix, in which diagonal elements are the degrees of each vertex and ${\tilde{D}}_{\mathrm{ii}}=\sum_{j}\;{\tilde{A}}_{\mathrm{ij}\;}$.

Once the atom embedding vectors $\left\{h_{i}^{c}\right\}$ of compound *c* are obtained, a readout operation finally turns them into the embedding vector of the compound. In the readout, both a global Max-pooling and a global Mean-pooling are performed in parallel. Their resulting embeddings, $Z_{\max}\left(j\right)=\underset{i}{\mathrm{argmax}}\;\{h_{i}^{c}(j)\}$ and $Z_{\mathrm{mean}}\left(j\right)=\underset{i}{\mathrm{mean}}\left\{h_{i}^{c}(j)\right\}$, are concatenated as the final embedding $Z\in R^{2t}$ of compound *c*.

### 2.3 Pathway encoder

The metabolic pathway encoder, containing a two-layer GCN architecture, learns pathway embedding representations by constructing a pathway dependence graph, which characterizes asymmetric pathway interdependence. In such a graph, nodes are pathways $T=\left\{t_{c},c=1,\ldots ,C\right\}$ and directed edges are their asymmetric dependences. A compound $c_{i}$ is assigned with a set of attending pathways $T_{i}\subseteq T$.

Inspired by Chen *et al.* (2019), the construction of the graph includes two phases, node initialization and edge building. The node initialization assigns initial node features. However, they cannot be directly learned in an end-to-end manner due to the small number of pathways (i.e. 11 pathways in the benchmark dataset). To address this issue, a pre-training strategy is adopted to obtain initial pathway embeddings. Considering that pathway names are semantic, we apply GloVe, a word representation tool, to implement the pre-training (Pennington *et al.*, 2014). For the pathway set, GloVe generates initial pathway embeddings $\left\{q\in R^{r}\right\}$ by learning word embeddings of semantic pathway names based on the Common Crawl dataset (https://nlp.stanford.edu/projects/glove/).

The edge building contains three steps to capture the pathway interdependence. First, the pathway co-occurrence is calculated based on the dataset in hand. Define the co-occurrence matrix as $U\in R^{C\times C}$, where *u_i,j_* is the pairwise co-occurrence counts between pathway *t_i_* and *t_j_*, *u_i,j_* = *u_j,i_* and *C* denotes the number of metabolic pathway types.

Then, a probability matrix **P** is calculated by the co-occurrence matrix U. Define *p_ij_* = *p* (*t_j_*|*t_i_*) as the probability of *t_j_* occurring when given *t_i_* occurring, and $N_{i}\;$as the total occurrence count of *t_i_*. The probability matrix *p_ij_* can be calculated by = *u_i,j_*/*N_i_* . Note that *P_ij_* is usually not equal to *P_ji_*.

As Chen *et al.* (2019) suggested, a binarization of **P** is performed to address the possible long-tail distribution of co-occurrence patterns, where a few {*P_ij_*}are significantly greater than others. Formally, the conditional probability matrix **P** is turned to a binary matrix${\;M=\{m_{ij},\;i,j=1,\ldots ,C\}\in R}^{C\times C}$ by a hard threshold τ as:

(5) mij= 0, if pij<τ 1, if pij≥τ.

This binarization removes trivial edges, which could be noise.

Last, to enhance the node distinguishability during the aggregation of neighboring nodes, an extra re-weighting scheme is utilized (Li *et al.*, 2018) as follows:

(6) $$m_{ij}^{w}=\frac{m_{ij}}{{\left(1*M\right)}_{j}},$$

where **1**  $\in R^{1\times C}$ and $M^{w}$ is the re-weighted dependence matrix.

Once the pathway interdependence graph is constructed, two layers of GCNs are used to learn pathway embeddings. Denote $q_{c}\in R^{r}$as the initial feature vector of a given pathway $t_{c}$ and $Q\in R^{C\times r}$ as the initial pathway feature matrix stacked by $\left\{q_{c},c=1,2,\ldots ,C\right\}$. Passing through a GCN layer, Q is updated by the following propagation rule:

(7) $$H_{x}^{\left(l+1\right)}=\sigma \left(D_{x}^{-1}{M_{x}H}_{x}^{\left(l\right)}\;W_{x}^{\left(l\right)}\right),\;l=0,1,$$

where $M_{x}={\alpha *M}^{w}+I_{C}$, $D_{x}\in R^{C\times C}$ is its degree matrix where ${\;d}_{\mathrm{ii}}=\sum_{j}\;M_{x}(i,j)$, $H_{x}^{\left(l\right)}\in R^{C\times r}\;$is the embedding matrix in the lth layer, α∈[0,1] is a trade-off coefficient, which determines how neighboring nodes are emphasized in the convolutional aggregation.

In addition, $I_{C}$ is the identity matrix, σ(·) denotes an LeakyReLU activation function and $W_{x}^{\left(l\right)}$ is a transformation weight matrix to be learned. Specifically, the input pathway feature matrix $H_{x}^{\left(0\right)}=Q$, while the output of the second layer $H_{x}^{\left(2\right)}$ is just the pathway embedding feature matrix, denoted as $O\in R^{C\times B}$, where *B* is the dimension of pathway embedding features. The dimension can be same as that of compound embedding features for the further purpose of measuring the proximity between pathways and compounds.

### 2.4 Multi-label predictor

After obtaining compound embeddings and pathway embeddings, we can directly perform discriminate the pathway set, in which a compound could attend. Inspired by Chen *et al.* (2019), we measure the proximities between a given compound and a list of pathways as follows:

(8) $$\hat{Y}=Oz,$$

where $z\in R^{B\times 1}$ is the embedding vector of a compound *c* and $O\in R^{C\times B}$ is the pathway embedding feature matrix, of which each row denotes the embedding of pathway *j*. The proximity ${\hat{y}}_{i}$ is the predicting score of the given compound attending in the *i*-th pathway among the pathway list T.

However, such a direct proximity measure would be senseless since the compound embedding space and the pathway embedding space are of different vector spaces. To address this issue, we design an adapter to map the compound embedding space into the pathway embedding space. The adapter can be implemented by a dense neural network (DNN) containing an input layer, a hidden layer, and an output layer. Thus, the final compound representation feature is defined as $z=\mathrm{DNN}(z)\in R^{B}$.

Last, the multi-label classification loss (He *et al.*, 2021) is used when training MLGL-MP. It is defined as follows:

(9) $$\mathrm{Loss}=-\frac{1}{C}*\sum_{i=1}^{c}y_{i}*\log\left((1+\text{exp}\;(-{\hat{y}}_{i}){)}^{-1}\right)+(1-y_{i})*\log\left(\frac{\;\text{exp}\;(-{\hat{y}}_{i})}{\left(1+\text{exp}\;(-{\hat{y}}_{i})\right)}\right),$$

where $y_{i}\in \{0,\;1$} are a true label indicating whether or not the compound participates in pathway *i*, and ${\hat{y}}_{i}$ is the corresponding confidence score output by MLGL-MP.

### 2.5 Evaluation metrics

To evaluate the performance of multi-label learning models, we follow the conventional settings in Baranwal *et al.* (2020) and Yang *et al.* (2020), which use Accuracy, Precision, Recall and F1_score as the performance metrics. The greater value these metrics are, the better performance the model achieves.

Furthermore, we use four additional indicators designated for multi-label learning (Paniri *et al.*, 2020; Zhang *et al.*, 2019), including Hamming Loss (HL), Ranking Loss (RL), Coverage and One Error (OE). HL provides an assessment how many times a pair of sample label is misclassified. RL provides an assessment about the fraction of reversely ordered label pairs. Coverage provides an assessment how far the list of ranked labels goes down to cover all the truth labels of samples on average. OE provides an assessment about the fraction of samples whose top-ranked label is not in the set of proper labels. For HL, RL, Coverage and OE, the smaller the values, the better the performance.

## 3 Experiments

### 3.1 Dataset

The experimental dataset was taken from Baranwal *et al.* (2020), which includes 6669 compounds and their metabolic pathway entries. The dataset was originally collected from KEGG Pathway (https://www.genome.jp/kegg/pathway.html), which contains 11 types of metabolic pathways, including (i) Carbohydrate metabolism; (ii) Energy metabolism; (iii) Lipid metabolism; (iv) Nucleotide metabolism; (v) Amino acid metabolism; (vi) Metabolism of other amino acids; (vii) Glycan biosynthesis and metabolism; (viii) Metabolism of cofactors and vitamins; (ix) Metabolism of terpenoids and polyketides; (x) Biosynthesis of other secondary metabolites; and (xi) Xenobiotics biodegradation and metabolism.

**Fig. 3.**
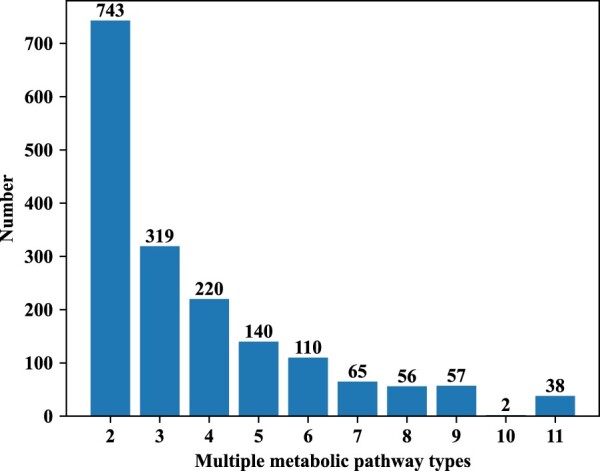
Compound distribution on multiple metabolic pathways

As the compound encoder of our MLGL-MP requires, we removed 21 compounds that cannot be converted to molecular graphs. We finally constructed a dataset containing 6648 compounds and their 11435 metabolic pathway entries. Among them, 4898 compounds attend only one metabolic pathway while the remaining 1750 compounds attend multiple metabolic pathways. Specifically, 38 out of 1750 compounds attend all the 11 metabolic pathways. The metabolic pathway dataset is summarized in Table 1 and in Fig. 3. More details can be found at https://github.com/dubingxue/MLGL-MP.

**Table 1.** Statistics of metabolic pathway dataset

Type	Metabolic pathway types	Involving compounds
*l_0_*	Carbohydrate metabolism	1126
*l_1_*	Energy metabolism	750
*l_2_*	Lipid metabolism	1066
*l_3_*	Nucleotide metabolism	342
*l_4_*	Amino acid metabolism	1440
*l_5_*	Metabolism of other amino acids	597
*l_6_*	Glycan biosynthesis and metabolism	325
*l_7_*	Metabolism of cofactors and vitamins	948
*l_8_*	Metabolism of terpenoids and polyketides	1483
*l_9_*	Biosynthesis of other secondary metabolites	1906
*l_10_*	Xenobiotics biodegradation and metabolism	1452

### 3.2 Parameter setting

In the compound encoder, each node of input compound graph was initially represented by a 78-dimensional (78-d) binary atom feature vector, as suggested in Nguyen *et al.* (2021). In brief, the initial representation of a compound contains five groups of atom indicators, including the atom symbol (44-d), the number of adjacent atoms (11-d), the number of adjacent hydrogens (11-d), the implicit value of the atom (11-d) and the atom occurrence in an aromatic structure (1-d). More details can be found in DeepChem (Ramsundar, 2018). Moreover, its GAT layer adopted 10 heads of attention layers, of which the outputting atom dimension was also set as 78 to capture the importance of chemical bonds. Similarly, its GCN layer kept the same dimension (780) of outputting atom features as that in the GAT layer. Finally, through the Global Max-pooling and the Global Mean-pooling on atoms, each compound was represented a 1560-dimensional embedding vector.

For the pathway encoder, the pre-training of 11 metabolic pathways was implemented by the GloVe algorithm (Pennington *et al.*, 2014), which represent their names in 300-dimensional embedding vectors based on the Common Crawl dataset containing 840 billion tokens and 2.2 million vocab. Both the two layers of GCNs in the pathway encoder were represented 1024-dimensional node embeddings. Finally, each pathway was represented as a 1024-dimensional vector.

In the multi-label predictor, the adapter was implemented by a DNN, of which the input layer, the hidden layer and the output layer contain 1560, 1500 and 1024 neurons, respectively.

**Fig. 4.**
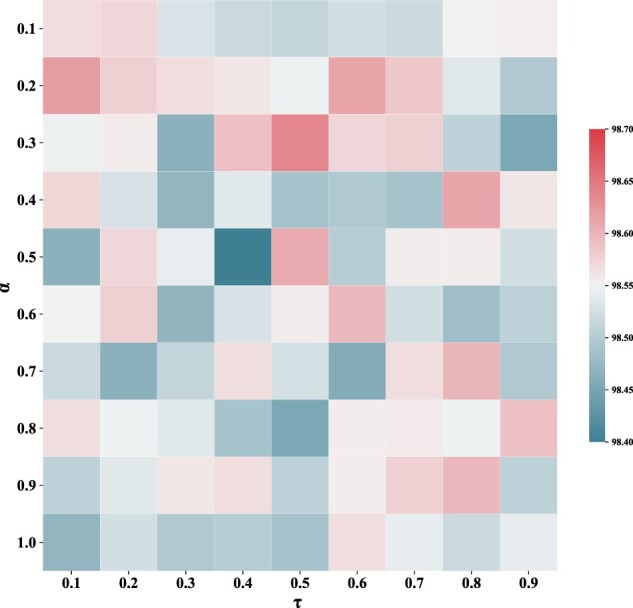
Grid search expanded by τ and α in terms of accuracy

After setting up the architecture of MLGL-MP, we investigated how its hyperparameters, including the threshold τ in Equation (5) and the coefficient α in Equation (7) influence the metabolic pathway prediction. For the pathway dependence matrix, we tuned the value of τ for the list of {0.1, 0.2, 0.3…,0.9}. We discarded two non-convergence cases where τ=0 indicates no edge removed and τ=1 generates a zero-dependence matrix. Furthermore, we set α in a set of {0.1, 0.2, 0.3…,0.9, 1.0}. Similarly, we discarded the case of α=0, which makes the dependence matrix as an identity matrix. The Accuracy metric was adopted to evaluate the investigation generated by the grid research on τ and α. The results show that the pair of τ = 0.5 and α  =0.3 accounts for the best performance of MLGL-MP (Fig. 4). Also, we tuned the learning rate from the list {0.1, 0.01, 0.001, 0.0005, 0.0001}, where 0.0005 accounts for the best performance.

In addition to these fine-tuned hyper-parameters, we empirically set the number of epochs as 200, set the batch size as 256 and selected Adam as the optimizer. Similarly, ReLU and LeakyReLU with default parameters were used as the activation functions in the compound encoder and the pathway encoder, respectively.

All the remaining experiments were run under the optimal values of these parameters.

### 3.3 Comparisons with baselines

We assessed the performance of MLGL-MP by the comparison with two state-of-the-art shallow learning methods, (i.e. RF and XGBoost), as well as two state-of-the-art deep learning methods, including a GCN-based method (Baranwal *et al.*, 2020) and a GAT-based method (Yang *et al.*, 2020). They are briefly summarized as follows.

- RF-based model: Since RF was originally designed for multi-class classification but not multi-label classification, we implemented RF in the exact same way as that in Baranwal *et al.* (2020). Eleven RF classifiers were trained separately to recognize each pathway type with the parameter setting where the maximum depth of the tree is 60 and the number of decision trees is 300.
- XGBoost-based model: The implementation of XGBoost is similar to that of RF. Eleven XGBoost classifiers were trained separately with a parameter setting where the maximum depth of the tree is 30 and the number of decision trees is 300.
- The GCN-based model (Baranwal *et al.*, 2020): it proposes a compound subgraph representation learning based on GCNs and combined the learned subgraph embeddings as local features with global features (diverse molecular properties, MACCS fingerprints, adjacency matrix, etc.) to a feedforward neural network. We used the same parameters as those in the original paper.
- The GAT-based model (Yang *et al.*, 2020): it adopts GATs to obtain compound subgraph representation as local features and used the same global features same to the GCN-based model (Baranwal *et al.*, 2020). We used the default values of parameters as those in the original paper.

For a fair comparison, we utilized 10-fold cross-validation (10-CV) for all the methods and measured their performance by two groups of metrics (Table 2). The first group contains the average Accuracy, Precision, Recall and F1_score. The greater, the better. The second group contains HL, Coverage, OE and RL, which are designated metrics for multi-label learning. The smaller, the better. The results show that the deep learning-based methods (the GCN-based, the GAT-based and our MLGL-MP) outperform the shallow learning-based methods (the RF-based and the XGBoost-based) overall. Moreover, it reveals that MLGL-MP achieves the best performance with significant improvements over all the metrics, compared with the GCN-based model and the GAT-based model. Therefore, the comparison demonstrates the superiority of our MLGL-MP.

**Table 2.** Performance evaluation on the KEGG dataset of multi-label metabolic pathway prediction

Method	Accuracy (%)	Precision (%)	Recall (%)	F1_score (%)	HL	Coverage	OE	RL
RF	97.59 ± 0.19	83.58 ± 0.84	83.54 ± 0.79	83.56 ± 0.81	0.024 ± 0.002	1.809 ± 0.069	0.156 ± 0.008	0.167 ± 0.008
XGBoost	98.04 ± 0.18	89.66 ± 0.58	90.49 ± 0.85	90.07 ± 0.64	0.020 ± 0.002	1.447 ± 0.087	0.099 ± 0.005	0.100 ± 0.008
GCN-based	97.53 ± 0.41	91.37 ± 1.20	93.22 ± 1.60	92.28 ± 1.30	0.025 ± 0.004	1.033 ± 1.140	0.100 ± 0.153	0.040 ± 0.082
GAT-based	97.57 ± 0.18	92.71 ± 0.64	92.04 ± 0.87	92.53 ± 0.39	0.024 ± 0.002	0.830 ± 0.318	0.064 ± 0.082	0.024 ± 0.028
**MLGL-MP**	**98.64 ± 0.47**	**95.26 ± 2.25**	**94.21 ± 1.94**	**94.73 ± 1.89**	**0.014 ± 0.005**	**0.559 ± 0.113**	**0.050 ± 0.019**	**0.011 ± 0.003**

### 3.4 Ablation studies

**Fig. 5.**
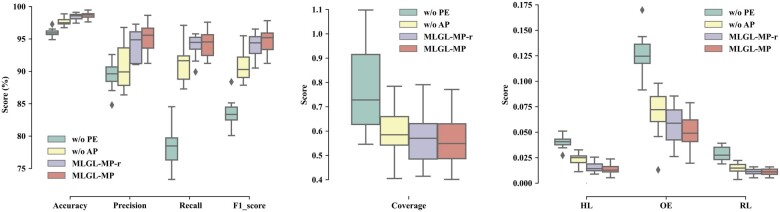
Ablation comparison. Compared with MLGL-MP, w/o PE removes the pathway encoder, w/o AP lacks the adapter in the multi-label predictor, MLGL-MP-r denotes alters the pre-trained node feature vectors in the pathway dependence graph to randomly initialized Gaussian vectors. The left panel indicates the performance by Accuracy, Precision, Recall and F1-score. The middle one indicates the performance with regard to Coverage. The right panel indicates the comparison in terms of Hamming Loss (HL), One Error (OE) and Ranking Loss (RL)

In this section, we investigated why MLGL-MP can achieve inspiring prediction by ablation studies. We made three variants of MLGL-MP, of which the first removes the pathway encoder (denoted as w/o PE), the second one lacks the adapter in the multi-label predictor (denoted as w/o AP), the third (denoted as MLGL-MP-r) alters the pre-trained node feature vectors in the pathway dependence graph to randomly initialized Gaussian vectors (Fig. 5).

MLGL-MP significantly outperforms w/o PE over all the evaluation metrics. In detail, compared with w/o PE, MLGL-MP improves the Accuracy by 2.70%, the Precision by 5.88%, the Recall by 15.86% and the F1-score by 11.27% due to its pathway encoder. Meanwhile, MLGL-MP reduces the HL by 0.027, the Coverage by 0.212, the OE by 0.076 and the RL by over 0.018. The result indicates that the designated pathway embeddings improve metabolic pathway prediction greatly because it captures the pathway interdependence.

Moreover, the comparison with w/o AP shows a similar improvement over all the metrics. In detail, MLGL-MP improves the accuracy by 1.01%, the precision by 4.43%, the recall by 3.16% and the F1-score by 3.84%. Again, it shows a better performance of multi-label learning with reducing the HL by 0.009, the Coverage by 0.046, the OE by 0.019 and the RL by 0.003. This result shows that the adapter in the predictor improves the prediction significantly by aligning compound embeddings with pathway embeddings.

In addition, the comparison shows that the version with pathway pre-training (MLGL-MP) is better than that with pathway random initialization (MLGL-MP-r) over all the evaluation metrics. Thus, the pre-training strategy can improve the prediction.

In general, the pathway encoder, the adapter and the pre-training strategy play indispensable roles in predicting multi-label metabolic pathways.

### 3.5 Case study: interpretability of MLGL-MP

Although deep learning is known as a black-box model, it is essential to understand how the model makes a prediction and whether the model can guide lead compound optimization in drug discovery. MLGL-MP leverages the GAT layer in its compound encoder to access why a compound participates in a specific pathway. Since the attention weights learned in the GAT layer can reflect the importance of chemical bonds in compounds, we can reveal the association between compounds’ substructures and their metabolic pathway.

**Fig. 6.**
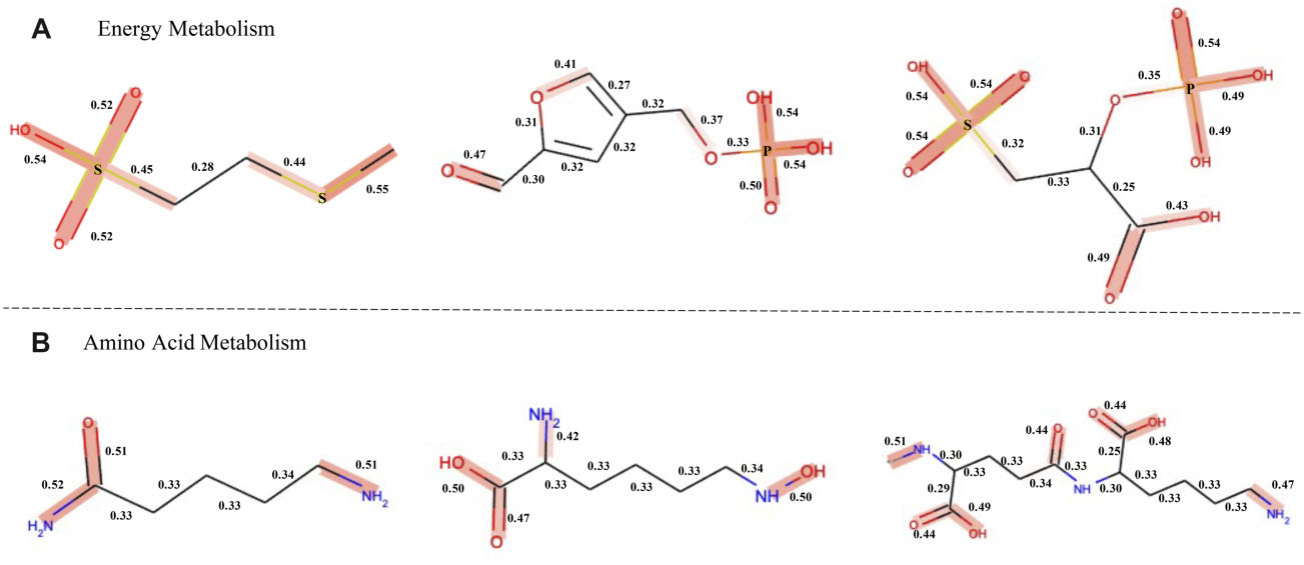
Visualization of compound substructure importance. (**A**) Energy metabolism; (**B**) Amino acid metabolisms.

For example, Energy Metabolism and Amino Acid Metabolism are two important pathways in organisms (Rui, 2014; Vettore *et al.*, 2020). The former maintains the regular activity of metabolic enzymes (Foo *et al.*, 2020; Motohashi and Akaike, 2019) while the latter are an essential process in cells (Lopez and Mohiuddin, 2021). Thus, we selected them as examples to illustrate the interpretability of MLGL-MP (Fig. 6).

Overall, the visualized attention weights show that most carbon (C)-based chemical bonds constructing compound backbones usually have small attention values. More importantly, the visualization reveals that crucial substructures having high attentions are pathway-specific. We went deeper into the case of Energy Metabolism (Fig. 6A), where Sulfur (S) and Phosphorus (P)-based chemical bonds in Energy Metabolism have higher attention values and are highlighted in red. The result is consistent with the early knowledge that *Sulfur metabolism* and *Oxidative phosphorylation* occur in the pathway of Energy Metabolism (Gibson and Skett, 2013). Meanwhile, recent works also provided more pieces of evidence (Foo *et al.*, 2020; Motohashi and Akaike, 2019). For example, the unique role of *sulfur* in organisms is mainly related to redox reactions and its functions include cell protection and energy metabolism (Motohashi and Akaike, 2019). *Oxidative phosphorylation* has garnered increasing interest in energy metabolism as a new target space (e.g. the mycobacterial druggable target) (Foo *et al.*, 2020). Moreover, the case of Amino Acid Metabolism (Fig. 6B) shows that the substructures of amino and carboxylic have greater attention weights. The result is validated by the work (Lopez and Mohiuddin, 2021), which indicates both *amino* (−NH2) and *carboxylic acid* (−COOH) functional groups play an important role in Amino Acid Metabolism.

In summary, MLGL-MP is an interpretable model, which can indicate compound substructures significantly associated with metabolic pathways. It would help reveal why a compound participates in a specific pathway.

## 4 Conclusion

In this paper, we have proposed an MLGL-MP, which contains a compound encoder, a pathway encoder and a multi-label predictor. This end-to-end framework can address two existing issues, including inadequate characterization of pathway dependences and interpretable prediction.

The comparison with popular shallow learning models and deep learning models demonstrates the superiority of MLGL-MP. Moreover, the ablation studies as well as the case study validate its contributions. First, it provides an interpretable manner to indicate crucial compound substructures which are significantly associated with metabolic pathways by molecular graph attention embedding. Secondly, by capturing the pathway interdependence, it significantly improves the characterization of the relevance between compounds and their metabolic pathways. Thirdly, by measuring the proximity between compounds and metabolic pathways in a common embedding space, it proposes a direct multipathway prediction approach without extra label strategy. In summary, we believe that our study provides new insights into label dependence representation learning for other multi-label classification problems (e.g. drug toxicity prediction) in drug discovery.

Moreover, though the GAT can interpret the importance of drug substructures to metabolic pathways in some sense, other parts (i.e. the pathway encoder and the adapter) in the model are of the black box. In the coming future, it is anticipated that interpretable techniques derived from image processing (e.g. visualization of hidden layers, nearest neighbors and GAN) can be utilized to achieve better interpretability in predicting metabolic pathways for compounds.

## Funding

This work has been supported by the National Nature Science Foundation of China [Grant number 61872297) and Shaanxi Provincial Key Research & Development Program, China (Grant number 2020KW-063).

*Conflict of Interest*: We declare that we have no conflict of interest.
